# Physicochemical and Functional Properties of Polyphenolic Core Gel Microcapsules from Rose Petals (*Rose* L.): A Comparative Study

**DOI:** 10.3390/foods15122134

**Published:** 2026-06-13

**Authors:** Natalia Żurek, Andżelika Padowska, Andżelika Kusy, Karolina Ochab

**Affiliations:** Department of Food Technology and Human Nutrition, Faculty of Technology and Life Sciences, University of Rzeszow, 4 Zelwerowicza St., 35-601 Rzeszow, Poland

**Keywords:** rose, microcapsules, polymer matrix, extract dose, physicochemical properties, functional properties

## Abstract

The aim of this study was to evaluate the effect of matrix type and dose of polyphenolic core from rose petals on the physicochemical and functional properties of microcapsules. Microcapsules were obtained by ionotropic gelation using four carrier systems: sodium alginate (SA), sodium alginate with added starch (SA + S), protein isolate (SA + P), and vegetable gum (SA + G). Polyphenolic compounds isolated from rose petals (E) were used as the core at six concentrations (0.25, 0.5, 1.0, 1.5, 2.0, and 2.5%). Differences between microcapsules were assessed based on physicochemical properties, polyphenol and anthocyanin content, antioxidant activity, swelling index, and biocompatibility. The results showed that both the extract dose and the matrix system significantly affected the analyzed parameters. The highest encapsulation efficiency was demonstrated for the lowest dose (0.25%), regardless of the matrix used. Total polyphenol and anthocyanin content significantly increased for all microcapsule versions with increasing extract dose, with the highest concentrations obtained for the SA + G system. These results strongly correlated with antioxidant activity and biocompatibility with human colonocyte membranes. In turn, the swelling index decreased with extract dose, showing the highest values in small intestinal fluid and the lowest in gastric fluid. These findings may have significant implications for the design of functional carriers for use in food and dietary supplement production.

## 1. Introduction

Microencapsulation is a form of immobilization of active ingredients within carrier shells. Several microencapsulation methods can be distinguished, including spray drying, coacervation, interfacial and in situ polymerization, emulsification, extrusion, and fluidization [1]. However, recently, microencapsulation through ionotropic gelation has been gaining popularity, mainly due to mild process conditions; the possibility of encapsulating sensitive compounds; the use of non-toxic, biocompatible materials; simplicity; and the low cost of the technology [2]. In this method, hydrogel microcapsules are formed as a result of polymer cross-linking by multivalent ions, such as Ca^2+^, which cause the polymer solution to transform into a gel and enclose the active substance in its structure [3]. Sodium alginate (SA) is one of the most commonly used polymers. However, the low efficiency of the encapsulation process for SA alone has been repeatedly demonstrated. Therefore, efforts are currently underway to increase the binding of active substances by the carrier matrix. This can be achieved by adding animal and plant proteins (e.g., whey protein isolate, pea protein isolate, soy protein isolate, zein protein isolate), plant gums (guar gum, xanthan gum, tara gum, arabic gum), and polysaccharides (pectin, starch, cellulose, carrageenan). The selection of the type and proportion of individual carriers plays a significant role in determining the efficiency of the process, but also influences the physicochemical properties, stability, core protection level, biocompatibility, solubility, active substance release kinetics, and health-promoting activity of the resulting microcapsules [3,4,5,6].

The level of these properties of microcapsules may also be determined by the concentration of the active substance constituting the microcapsule core. Vitamins, enzymes, probiotics, dyes, flavors, fragrances, oils, essential oils, and bioactive ingredients are most commonly used in this role [1,2]. The last group includes polyphenolic compounds, which are most abundant in plant-based ingredients. Flowers are a rich and diverse source, among which rose petals (*Rosa* L.) are particularly noteworthy. Compared to other types of edible flowers, rose flowers are the most widely known and most frequently used raw material in the food, cosmetics, and pharmaceutical industries [7]. On the one hand, this is due to the widespread cultivation of roses, and on the other hand, to the rich profile of bioactive compounds [8]. The total polyphenol content in rose flowers ranges from 5.21 to 427.6 mg GAE/100 g fresh weight [9]. The dominant polyphenolic compounds include anthocyanins, mainly cyanidin 3,5-di-*O*-glucoside (94.9% of all anthocyanins) and pelargonidin 3,5-di-*O*-glucoside [9]. Other polyphenols identified in rose petals include hyperoside, kaempferol-3-*O*-rutinoside and rutin [10]. The rich polyphenol composition of rose flowers translates into a wide range of health-promoting properties of this plant, including antioxidant, anti-inflammatory, antibacterial, hepatoprotective and cardioprotective effects [11,12].

However, despite the above-mentioned broad activity, polyphenols present in rose petals are compounds of low stability during storage, technological processing and digestion in the gastrointestinal tract [2,3]. One form of protection may be gel microencapsulation. Despite growing interest in the microencapsulation of bioactive compounds, the influence of the carrier matrix composition, particularly the degree of use of various polysaccharide–protein systems and the concentration of the polyphenolic core, on the physicochemical and functional properties of microcapsules remains poorly understood. Therefore, sodium alginate was chosen as the primary encapsulating material due to its ability to form stable hydrogels. Protein, vegetable gum, and starch were added to modify the matrix properties. These materials represent biopolymers with distinct functional properties that can influence the microcapsule structure and the stability of the encapsulated polyphenols. This selection will enable the assessment of the effect of the encapsulating matrix composition on the protection of polyphenolic compounds contained in the rose petal extract.

Therefore, the aim of this study was to evaluate the effect of matrix type and polyphenolic core dose on the physicochemical and functional properties of microcapsules. Microcapsules were obtained by ionic gelation using various carrier systems, including sodium alginate (SA), sodium alginate with added starch (SA + S), protein isolate (SA + P), and vegetable gum (SA + G), with a core composed of polyphenolic compounds isolated from rose petals (E), administered at six concentrations (0.25, 0.5, 1.0, 1.5, 2.0, and 2.5%). We believe that this research profile will provide missing knowledge on scaling the ionotropic microencapsulation process based on matrix composition and core dose, which will be important in the design of functional carriers for use in food and dietary supplement production.

## 2. Materials and Methods

### 2.1. Materials

Red rose (*Rose* L.) petals were purchased from Świat Herbat (Tarnobrzeg, Poland). Sodium alginate (SA) was purchased from Sigma-Aldrich (Steinheim, Germany). Potato starch was purchased from Wielkopolskie Przedsiębiorstwo Przemysłu Ziemniaczanego (Luboń, Poland). Pea protein (80% protein content) was purchased from Diet-food (Opatówek, Poland). Guar gum was purchased from Guiltfree (Otrębusy, Poland).

### 2.2. Isolation of Polyphenols

The polyphenol fraction from rose petals was isolated according to previous work [13]. Briefly, ground flowers were first subjected to exhaustive extraction by using methanol at increasing concentrations (50%, 70%, and 96%). Each extraction step was assisted by ultrasonication (Sonic 10 bath, Polsonic, Warsaw, Poland) for 20 min at 35 °C and 40 kHz. Each extraction step was separated from the filtrate using a centrifuge (type 5430, Eppendorf, Hamburg, Germany). The evaporated extract from the previous step was then applied to a glass column packed with C18 resin (LiChroprep RP-18, 40–63 µm (Merck KGaA, Darmstadt, Germany)). Compounds other than polyphenols were eluted with water, while the polyphenolic fraction was eluted with 96% methanol. The resulting filtrate was lyophilized into a powder (ALPHA 1–2 LD plus, Osterode, Germany).

### 2.3. Preparation of Microcapsules

Microencapsulation was performed using an encapsulator (B-390, BUCHI, Flawil, Switzerland). Carrier solutions were previously prepared: 2% sodium alginate (SA), 2% SA with 1% potato starch (SA + S), 2% SA with 1% pea protein (SA + P), and 2% SA with 1% guar gum (SA + G). Purified polyphenolic extract was added to the prepared carriers in six doses: 0.25, 0.5, 1.0, 1.5, 2.0, and 2.5% (*w*/*v*). The encapsulator was operated at the following settings: pressure 500 bar, nozzle diameter 350 µm, electrode voltage 650 V, and frequency 400 Hz. The obtained microcapsules were hardened for 20 min in 3.5% CaCl_2_ solution, washed with water and lyophilized.

### 2.4. Size Measurement

Microcapsules were observed and their diameters were measured using an optical microscope (Oxion Inverso, Euromex, Mataro, Spain). Images were recorded at 20× magnification. Before analysis, the optical system was calibrated using a standard micrometer. The captured images were analyzed using ImageJ Version 1.54i software (Wayne Rasband, National Institutes of Health, Bethesda, MD, USA). For each sample type, 30 randomly selected microcapsules were analyzed. The diameter of a single microcapsule was determined as the distance between its opposite edges, measured at the widest point of the image cross-section. Based on the obtained results, the mean microcapsule diameter and SD were calculated.

### 2.5. Dry Matter

The dry mass of the microcapsules was assessed using the oven drying method. Samples were dried at 105 °C for 1 h in a laboratory oven (ED 115, Binder, Tuttlingen, Germany). The dry mass content was determined based on the difference in sample mass before and after drying.

### 2.6. Hydration Properties

The evaluation of the hydration properties of the microcapsules included determining hygroscopicity, water solubility (WS), and water absorption index (WAI). Briefly, to assess hygroscopicity, the microcapsules were placed in a 75% humidity chamber for 7 days. After this time, the hygroscopicity level was determined based on the difference in sample weight before and after storage. For the WS and WAI assessment, the microcapsules suspended in water were mixed for 30 min at 700 rpm (Thermomixer, Eppendorf, Hamburg, Germany) and then centrifuged (15 min at 7500 rpm). The WS value was calculated as the ratio of the dry mass of solutes in the supernatant to the initial mass of the sample. The WAI was calculated as the ratio of the mass of the hydrated pellet after hydration to the initial dry mass of the sample.

### 2.7. Color Measurement

Color determination was performed using an UltraScan spectrophotometer (HunterLab, Reston, VA, USA). Measurements were performed in the CIE Lab* color space. Additionally, total color change (∆*E*) and chroma (Chroma) were determined.

### 2.8. Total Polyphenol Content and Total Anthocyanin Content

First, microcapsule extracts were prepared by suspending them in 50% methanol, homogenizing them (T18 Ultra-Turrax, IKA, Warsaw, Poland), and then subjecting them to ultrasonication for 20 min at 35 °C and 40 kHz. After this time, the extract was centrifuged and used for analysis.

Total polyphenol content (TPC) was determined spectrophotometrically according to the method described by Gao et al. [14]. Results were expressed as mg GAE (gallic acid)/g. Total anthocyanin content (TAC) was also determined spectrophotometrically using the method of Lee et al. [15]. The results were expressed as mg C3G (cyanidin 3-*O*-glucoside)/g.

### 2.9. Encapsulation Efficiency

The efficiency of the encapsulation process was calculated based on the total polyphenol content (TPC) in the purified rose flower powder (W0) and the total polyphenol content in the microcapsules (W1):

$$EE\;\left(\%\right)=\;\frac{W1}{W0}\;\times \;100$$

### 2.10. Antioxidant Activity

Antioxidant activity was assessed using the ABTS^•+^ radical scavenging activity (ABTS) method, copper ion reduction (CUPRAC) method, and superoxide anion radical scavenging (O_2_^•−^) method. A detailed description of these assays can be found in our previous study [16]. The results for the ABTS and CUPRAC methods were expressed in mmol TE (Trolox)/100 g. The results for O_2_^•−^ were expressed as IC_50_ value (μg/mL).

### 2.11. Swelling Experiments

The swelling index of microcapsules was assessed in three fluids simulating human gastric fluid (SGF, pH 1.2), intestinal fluid (SIF, pH 6.8), and colonic fluid (SCF, pH 7.4). Solutions were prepared in phosphate buffer, with pH adjusted with 0.1 M NaOH or 0.1 M HCl. Weighed microcapsules were incubated in the prepared fluids maintained at 37 °C. The degree of microcapsule swelling was assessed at five time points (0.5, 1, 3, 5, 8 h), and the obtained results were presented as percentage weight increase (% swelling).

### 2.12. In Vitro Biocompatibility

The biocompatibility of the microcapsules was assessed using a normal human colonocyte cell line (CCD841 CoN). Cells were seeded in a 6-well plate (1 × 10^5^ cells/well) and grown to full confluence. Microcapsules suspended in DMEM culture medium were then applied to the formed membrane and left in contact for 48 h. After this time, the cells were washed twice with phosphate buffer, and cell viability was assessed according to the MTS assay (Promega, Madison, WI, USA). Results were expressed as percentage cell viability compared to the control (untreated cells).

### 2.13. Statistical Analysis

The assays were performed in triplicate. Results are presented as means and SD. Statistical analysis was performed using Duncan’s test, Student’s *t*-test, and principal component analysis (PCA) using Statistica 13.3 (StatSoft, Krakow, Poland).

## 3. Results and Discussion

### 3.1. Physicochemical Properties

**Figure 1:**
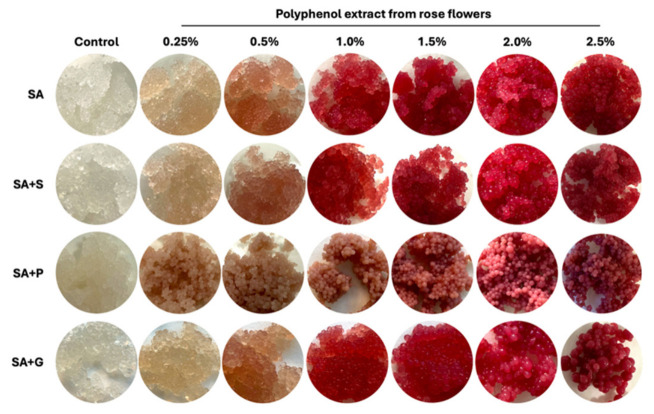
Photos of alginate (SA), alginate–starch (SA + S), alginate–protein (SA + P) and alginate–guar (SA + G) microcapsules with different percentages of polyphenolic extract from rose petals (E0.25–2.5%).

**Figure 2:**
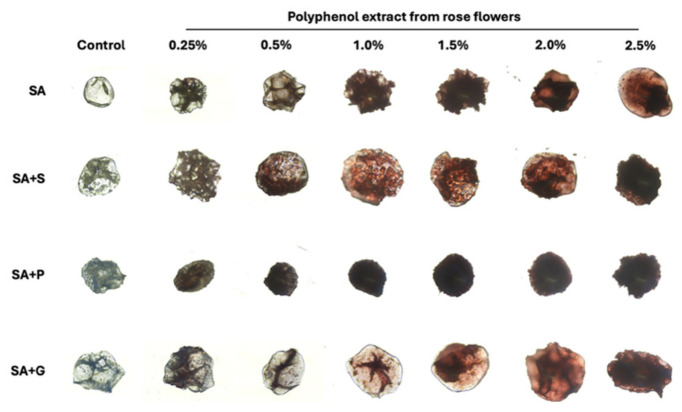
Microscopic images of alginate (SA), alginate–starch (SA + S), alginate–protein (SA + P) and alginate–guar (SA + G) microcapsules with different percentages of polyphenolic extract from rose petals (E0.25–2.5%).

Physicochemical analysis of alginate (SA), alginate–starch (SA + S), –protein (SA + P), and –gum (SA + G) microcapsules containing various doses of rose petal polyphenol extract included measurements of diameter, dry weight, hydration properties, and color. In the case of microcapsules, these parameters determine their suitability for consumption and their potential use as a functional food additive. Polyphenol extract concentrations higher than 2.5% led to complete deformation of the microcapsules during the encapsulation process; therefore, higher concentrations were not tested (Figure 1 and Figure 2).

The diameter of microcapsules is a fundamental parameter determining their industrial application. In our study, the diameter of microcapsules ranged from 388.8 to 990.5 μm (Table 1). This parameter depended on both the carrier composition and the concentration of the polyphenolic extract. Analyzing the matrices used, the lowest values were obtained for SA + P microcapsules (353.4–469.2 μm), and the highest for SA + G (887.5–994.6 μm). In turn, considering the extract dose, the lowest diameter was found for microcapsules with 0.5% extract addition (481.2–913.9 μm), and the highest with 2.5% addition (469.2–990.5 μm). The values shown were statistically significantly different (*p* < 0.05). These data are comparable to other studies using the gel encapsulation method [4,6]. In the case of the microencapsulation method used in this work, the microcapsule diameter is largely dependent on the selected encapsulator nozzle diameter. Other factors that can influence this parameter include the type of carrier, its concentration, the solution flow rate, and physical conditions such as temperature, Ca^2+^ ion concentration, and gelation time [17]. However, analyzing the physicochemical properties of the carriers used in this study, it can be assumed that the viscosity of the systems used had a significant impact on the microcapsule diameter. The addition of starch, protein, and gum to the SA may have affected the droplet formation process by increasing flow resistance and reducing shear forces on the outflowing stream. Consequently, the hindered breakdown of the carrier stream into smaller droplets led to the formation of microcapsules with a larger diameter [4]. Among the additives used, the gels based on guar gum are characterized by the highest viscosity, which resulted in obtaining microcapsules with the largest diameter [18]. The influence of the system viscosity on the size of microcapsules was also demonstrated by other authors [6,19].

Dry matter represents the actual amount of material remaining after water evaporates. However, higher values of this parameter determine product durability by limiting degradation reactions and microbial growth, improving microbiological quality and potentially extending shelf life [20]. In this study, the dry mass of microcapsules strongly correlated with the dose of polyphenolic extract and the composition of the carriers. In each of the four matrix versions, the value of this parameter increased with increasing extract addition. Furthermore, the highest ranges of dry mass were recorded for SA-G microcapsules (92.8 (SA + G)–96.0% (SA + G/E2.5)), and the lowest for SA microcapsules (90.0 (SA)–93.8% (SA/E2.5)). However, no statistically significant differences were found for SA + P, SA + P/E0.25 or SA + P/E0.5 microcapsules (*p* < 0.05). It has been previously proven that gels based on guar gum form a more compact and ordered structure, which consequently leads to an increase in dry mass content [21]. In future work, a higher addition of other matrices to the SA carrier should be considered, which would contribute to an increase in the value of this parameter and thus improve the microbiological quality.

Hydration properties determine the ability of microcapsules to absorb and bind water, which plays a significant role in maintaining their stability, functionality, and potential industrial applications. These properties were assessed by measuring hygroscopicity, water solubility (WS), and water absorption index (WAI). Microcapsule hygroscopicity decreased with increasing polyphenol addition. The highest values were observed for the SA + G carrier systems (72.1 (SA + G)–42.1% (SA + G/E2.5)), while the lowest values were observed for the SA + P carrier systems (41.6 (SA + P)–23.0% (SA + P/E2.5)). The same relationship was observed for the WAI parameter, with the highest values for the SA + G system (4.7 (SA + G)–2.7 (SA + G/E2.5)) and the lowest for SA + P (2.8 (SA + P)–2.2 (SA + P/E2.5)). However, an inverse relationship was observed for the WS parameter, where the values increased with increasing extract concentration. However, similarly to the previous two parameters, the highest water solubility values were shown for the SA + G system (29.3 (SA + G)–42.1% (SA + G/E2.5)), and the lowest for SA + P (16.7 (SA + P)–21.1% (SA + P/E2.5)). In the case of hygroscopicity and WAI, the increase in polyphenol concentration could have influenced the formation of additional bonds with the polymer matrix, which contributed to the decrease in the values for these two parameters. In turn, the WS parameter increased due to the dispersing and surfactant properties of polyphenols. The highest values of these parameters were observed in the SA + G system, most likely due to its high viscosity and ability to form a compact, hydrophilic network, while the lowest values were observed in the SA + P system due to the formation of impermeable polyphenol–protein complexes [22]. In the study by Xu et al. [23], the analysis of the solubility of starch–polyphenol powder from edible rose petals showed that the addition of the extract resulted in a decrease in starch solubility due to the reduced escape of amylose and amylopectin from starch.

Product color is one of the key parameters determining consumer acceptability. Differences in microcapsule color were more dependent on the degree of extract addition than on the type of carriers used. In each microcapsule variant, the L* parameter decreased with increasing extract addition, indicating darkening of the material. However, the a* parameter values increased, indicating a color shift toward red, while the b* parameter values decreased, indicating a decrease in the yellow content and a shift toward blue. Together, these changes suggest a shift in microcapsule color toward red-purple with increasing extract addition. The observed differences in the color parameters of microcapsules obtained from four different carriers and containing different concentrations of rose petal extract are directly related to the composition and concentration of the extract’s coloring compounds. The main group of pigments in rose flowers are anthocyanins, which are responsible for the red, violet, blue-violet, orange-red, light red and purple shades of their petals [24]. The dominant anthocyanins of rose flowers are cyanidin derivatives, mainly cyanidin-3-*O*-glucoside (47% of all anthocyanins), as well as malvidin glycoside (32%) and pelargonidin glycoside (18%) [9]. Moreover, purification of extracts from ballast substances contributed to a significant increase in the concentration of anthocyanins in the preparation mass, which was directly reflected in significant (*p* < 0.05) changes in the values of the L*, a* and b* parameters with the addition of the extract [4]. This hypothesis is supported by the values obtained for the Chroma (color saturation) and Δ*E* (total color difference with respect to free rose petal extract). Chroma increased with increasing extract addition, indicating greater color intensity and purity. However, Δ*E* values decreased with increasing polyphenol addition, exceeding a threshold of 5, indicating a color difference between samples visible to the naked eye (Figure 1). However, only from an extract concentration of 2% (for SA; SA + P; SA + G microcapsules) can a color change be observed, approaching that of pure rose extract. Therefore, it can be concluded that the selected carrier compositions can be used as carriers for pigments present in rose petals.

### 3.2. Encapsulation Efficiency

Encapsulation efficiency is a measure of the process’s effectiveness. This parameter is largely dependent on the concentration of the extract used to form the microcapsule core. Therefore, in this study, we assessed the effect of extract dose on gel encapsulation efficiency using various matrix systems.

**Figure 3:**
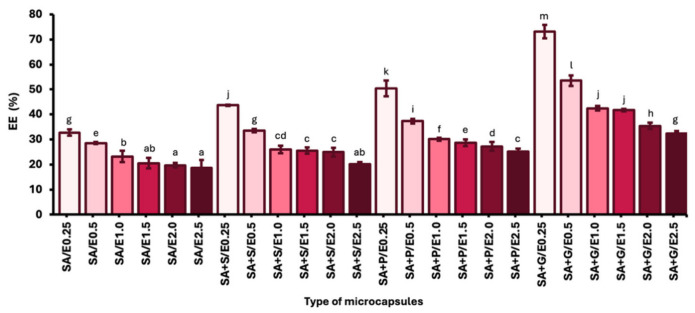
Encapsulation efficiency for alginate (SA), alginate–starch (SA + S), alginate–protein (SA + P) and alginate–guar (SA + G) microcapsules with different percentages of rose petal polyphenolic extract (E0.25–E2.5%). Results are presented as mean and SD. Values marked with different symbols (a–m) differ statistically significantly (*p* < 0.05).

As can be seen in Figure 3, with the increase in the amount of rose petal extract added, the encapsulation efficiency decreased, showing the highest values for the SA + G matrix (73.1 (SA + G/E0.25)–32.3% (SA + G/E2.5)), and the lowest for the SA matrix (32.7 (SA/E0.25)–18.6% (SA/E2.5)). The obtained values are consistent with the previous reports on the encapsulation of plant extracts in gel matrices [3,4,6,19]. At the same time, the cited works show a significant dependence of encapsulation efficiency on the concentration and type of polymer, the concentration of the active substance, the type and concentration of the curing agent, the gelation time, and the size of the microcapsules. Therefore, analyzing the carriers used in this work, the SA matrix is composed of G blocks that form stable ionic bridges, enabling the binding of polyphenols through hydrogen and electrostatic interactions [5]. However, compared to the other three matrices, the number of functional groups is the smallest, which can explain it having the lowest encapsulation efficiency. Many publications have shown that encapsulation with SA solution alone translates into low process efficiency [3,4,25].

We assume that the SA + S system promoted better binding of polyphenols through additional hydroxyl groups and hydrogen interactions between the hydrogen groups of both polysaccharides, which strongly retained polyphenols. Hassan et al. [19] demonstrated that the introduction of 0.5% corn starch into SA increased the nisin encapsulation efficiency by 1.4-fold (to 33.3%). At the same time, the cited authors proved that higher starch concentrations lead to a decrease in the value of this parameter. However, Gheorghita et al. [6] tested the encapsulation efficiency of metformin in different proportions of SA and wheat starch and showed the highest value (29.6%) for the system used in this work (2% SA + 1% S). This requires further research to confirm the existing relationships.

In turn, the polymer network composed of SA + P, where electrostatic interactions between the negatively charged carboxyl groups of SA and the positively charged amino groups of proteins stabilize the structure by effectively binding polyphenols [5]. Moreover, proteins have the ability to strongly bind polyphenols through hydrophobic and hydrogen interactions, especially with their hydroxyl groups, which may explain the higher encapsulation efficiency compared to the SA and SA + S matrix [26]. Also Żurek et al. [4] tested the effect of proteins of different origin on the encapsulation efficiency of polyphenols isolated from cranberries and proved a 1.1- to 1.4-fold increase in this parameter by introducing 1% of protein into the SA solution. The differences between the proteins used resulted from their amino acid composition, and the presence of reactive groups of amino acids arginine and leucine in pea protein may determine the formation of hydrogen and hydrophobic bonds with polyphenols [4,27,28].

The structure of the SA + G matrix allowed for the most effective binding of polyphenols as a result of the formation of a highly viscous system with a dense, hydrophilic and less porous structure with an additional large number of hydroxyl groups forming hydrogen bonds with polyphenols [5]. Ultimately, this leads to increased retention and stability of polyphenolic compounds. This has also been demonstrated in other studies, where the introduction of plant gums into the SA matrix resulted in increased encapsulation efficiency [29,30].

Analyzing the effect of the extract dose, the decrease in encapsulation efficiency with increasing extract concentration may result from the limited capacity of polysaccharide and polysaccharide–protein matrices, as well as from the increased diffusion of polyphenolic compounds into the hardening solution and from the disruption of the gel network formation process at higher polyphenol concentrations [5,27,28]. Previous studies have demonstrated the highest encapsulation efficiency at significantly higher concentrations of bioactive compounds used as the microcapsule core. For galactogogue herbal extract tested in the range of 1.0–5.0% and pomegranate peel extract administered in concentrations of 0.25–2.5%, the highest encapsulation efficiency values were obtained with the addition of 1% extract using SA solution as the matrix [31,32]. This difference compared to the present study may be due to the form of extract used as the microcapsule core. In our study, we used an extract purified from ballast substances, composed primarily of polyphenols, which is associated with a higher availability of reactive polyphenols. In the case of crude extracts, on the other hand, the higher presence of sugars, proteins, and other accompanying components may limit the availability of free polyphenols by partially binding them and increasing the viscosity of the system, which in turn reduces their interaction with the gel matrix and allows for the use of higher extract additives [5].

Based on the above results, it can be concluded that the system that provides the highest degree of protection and stabilization of polyphenolic compounds is the SA + G matrix with 0.25% addition of rose petal extract.

### 3.3. Total Polyphenolic and Anthocyanin Content

Polyphenolic compounds are among the main bioactive components found in rose petals. Their content is primarily correlated with the health-promoting properties of rose flowers. However, polyphenolic compounds are among the least stable groups of plant-derived compounds, which significantly limits their properties. Encapsulation is a method that can increase their stability and preserve their bioactivity [1,2].

The content of polyphenolic and anthocyanin compounds in rose petal powder was 387.0 mg GAE/g and 32.1 mg C3G/g dm, respectively (Table 2). These values are significantly higher compared to previously reported works [24,33]. This difference is primarily due to the preparation method. The use of solid-phase extraction allowed for the removal of ballast substances from the extract, yielding a preparation with a concentrated polyphenol content in a small mass. These conclusions have been repeatedly confirmed in our previous work [13,34].

Analyzing the content of polyphenols and anthocyanins in the obtained microcapsules, it can be seen that the amount increased proportionally to the extract dose used, and was significantly dependent on the type of carrier material. The best carrier for binding both polyphenols and anthocyanins was the SA + G matrix, where the content of these two groups of compounds ranged from 26.2 to 80.1 mg GAE/g and from 2.3 to 7.2 mg C3G/g, respectively. The remaining carriers, in terms of the analyzed parameters, can be ranked in the following order: SA + P > SA + S > SA. Polymer binding of polyphenols and anthocyanins depends on the ability to form hydrogen bonds, hydrophobic bonds, and electrostatic bonds, determined by the chemical structure and surface properties of the matrices used, as described in detail in the chapter on encapsulation efficiency (Section 3.2).

The effect of microencapsulation on the content of polyphenols and anthocyanins isolated from rose petals has not been studied to date. However, of these two groups of compounds, anthocyanins, primarily their cationic flavylide form, are rapidly degraded by external factors [35]. Moreover, previous studies have shown that during the gel encapsulation process, significant loss of these compounds occurs when curing in a CaCl_2_ solution [35,36]. Our research also revealed a decrease in the content of these compounds in microcapsules compared to pure extract. At the same time, other microcapsule studies have shown that enriching the SA matrix with other ingredients, such as whey protein isolate or pectin, leads to increased anthocyanin stability [3]. The same relationship can be observed in our studies. The penetration of anthocyanins into the curing solution is largely determined by the porosity of the microcapsules. Introducing additional carriers composed of carboxyl or amine groups into the SA matrix reduces the porosity of the resulting hydrogel structure, limiting the diffusion of anthocyanins [37]. Therefore, the best matrix binding polyphenols and anthocyanins was the SA + G system. Taking into account the encapsulation efficiency, the dose ensuring the highest stability of these compounds was the concentration of 0.25%.

### 3.4. Antioxidant Activity of Microcapsules

Antioxidant activity is an important element in the quality assessment of products due to the ability of compounds exhibiting this activity to inhibit oxidation processes and thus influence product shelf life, as well as their potential beneficial health-promoting effects in the human body, including neutralization of free radicals. Therefore, in this study, antioxidant activity was measured using the ABTS and CUPRAC methods, which reflect antioxidant activity in a chemical model, and the O_2_**^•−^** radical scavenging method, which is one of the reactive oxygen species generated in the human body.

For pure rose petal extract, the values for these three methods were 908.4, 321.2 mmol TE/100 g, and 87.4 μg/mL, respectively. Previously, using the CUPRAC method, a value of 0.5 mmol TE/100 g dw was obtained for rose petal extract [24]. In other works, a strong correlation between antioxidant activity and the total content of polyphenols and anthocyanins in the extract of various types of petals has been repeatedly demonstrated [9,38]. Hence, for the obtained microcapsules, as in the case of total polyphenol and total anthocyanin content, antioxidant activity increased proportionally to the extract dose and depended on the type of matrix used. It can therefore be assumed that the interactions between polyphenolic compounds and polymers, described in Section 3.2, also influenced the analyzed parameter. The highest values for the ABTS, CUPRAC, and O_2_**^•−^** methods were recorded for microcapsules with the SA + G/E2.5 matrix and amounted to 133.3, 46.7 mmol TE/100 g, and 428.4 μg/mL, respectively. Compared to the SA/E2.5 matrix, these values were 1.4-, 1.8-, and 1.2-fold higher, respectively. In our previous studies on the encapsulation of blueberry polyphenolic fraction using SA and SA with the addition of WPI, pectin and a WPI/pectin mixture, we proved that enrichment of the matrix with additional polymers led to a 1.8- to 2.4-fold increase in antioxidant activity (for the ABTS method), and the obtained results additionally correlated with the anthocyanin content and encapsulation efficiency. This was also confirmed in the studies of Stach et al. [39] and Ćorković et al. [36] for the encapsulation of anthocyanin-rich extracts in SA, pectin, chitosan and carrageenan matrices.

In general, enrichment of SA with additional polymers may be a valuable technique for preserving the antioxidant activity of extracts rich in polyphenolic compounds, which may be of great importance for the food and pharmaceutical industries.

### 3.5. Microcapsule Swelling Index

The swelling index allows us to define the degree of water absorption and structural changes in microcapsules during digestion in the gastrointestinal tract, which directly impacts their effectiveness as carriers of active substances. Therefore, this study analyzed the swelling behavior of microcapsules in phosphate buffers at pH 1.2, 6.8, and 7.2 at 37 °C, which corresponds to the conditions found in the stomach, small intestine, and colon.

**Figure 4:**
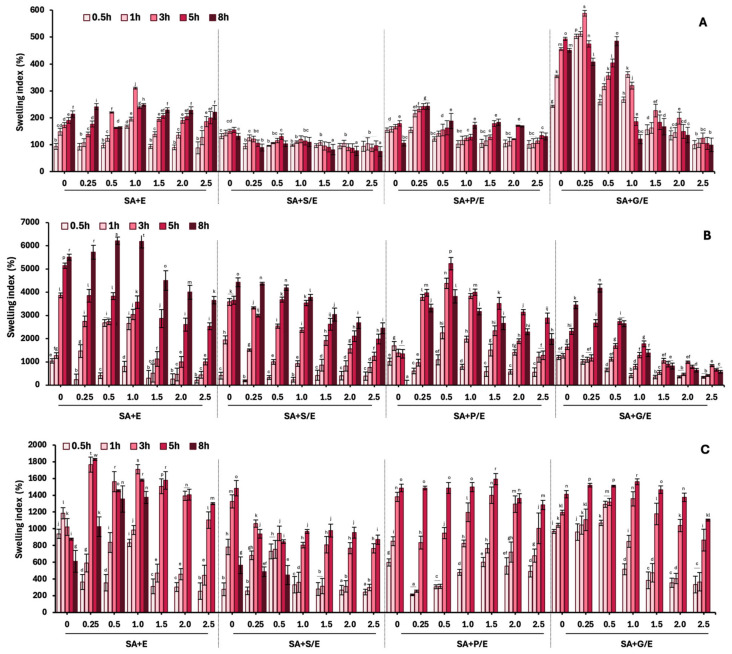
Swelling index in gastric (**A**), small intestine (**B**) and large intestine (**C**) fluid of alginate (SA), alginate–starch (SA + S), alginate–protein (SA + P) and alginate–guar (SA + G) microcapsules with different percentages of rose flower polyphenolic extract (E0.25–2.5%). Values marked with different symbols (a–w) differ statistically significantly (*p* < 0.05).

As can be seen in Figure 4, the swelling capacity of microcapsules depended primarily on their storage environment. The lowest swelling index was observed for microcapsules in a fluid simulating gastric juice, while the highest was observed in the small intestine and colon. These results are closely related to the physicochemical properties of the matrices chosen as carriers for the polyphenolic extract from rose petals. In an acidic environment, in the structure of SA—the main component of microcapsules—protonation of carboxyl groups occurs, as a result of which the soluble form of SA is transformed into alginic acid, which is poorly soluble [40]. This process results in a densification of the microcapsule structure and shrinkage, which explains it having the lowest swelling index values. The addition of starch and protein to SA can lead to the formation of hydrogen bonds (SA + S) and electrostatic complexes (SA + P), reducing the microcapsules’ ability to relax and swell [4,23]. In turn, a different relationship can be observed for the SA + G matrix, a strongly hydrophilic system that binds water well, where, compared to the SA matrix, the swelling index increased 2.6-fold during the 0.5 h incubation time. According to Seeli et al. [41], the increased swelling degree of the SA + G carrier can be explained by the presence of ionized carboxyl groups, the mutual electrostatic repulsion of which leads to an increase in the distance between the polymer chains and a loosening of the network structure.

However, the above-mentioned properties of the carriers used in the gastric fluid environment are altered by the presence of polyphenols. In most of the analyzed systems, maximum swelling was observed at low polyphenol concentrations, such as 0.25% for the SA + P and SA + G systems, and 1% for the SA system, while further increases in their content led to a decrease in this parameter. The exception was the SA + S matrix, where a gradual decrease in swelling was noted across the entire concentration range (0.25–2.5%). In one study, the decrease in starch swelling capacity after the introduction of polyphenolic extract from edible rose hips was attributed to oxidative cross-linking processes and interactions leading to the formation of stable complexes between polyphenols and amylopectin chains, which resulted in the formation of more complex structures with increased water-binding capacity while simultaneously limiting water availability to starch granules and reducing swelling [23]. For the remaining systems, the initial increase in swelling can be attributed to the loosening of the polymer network and an increase in its water-binding capacity [42,43]. However, at higher concentrations of polyphenols, hydrogen interactions dominate and, in protein systems, the formation of polyphenol–protein complexes leads to the thickening and stiffening of the gel structure and, consequently, to a reduction in its water absorption capacity [4,44].

Compared to the gastric fluid environment, a dramatic increase in microcapsule swelling can be observed in intestinal and colonic fluids. The properties of the main component, SA, again significantly impact the effect achieved. At pH 6.8–7.2, it transforms from alginic acid to its ionized form (-COO^−^), which causes chain repulsion, network loosening, and increased gel porosity [40,44]. Analyzing Figure 4B, it can also be seen that in intestinal fluid, the swelling ratio maximized at a specific polyphenol dose, followed by a decrease. This is also visible in colonic fluid (Figure 4C); however, in this environment, these changes are not as rapid as in the previous two fluids. In the small intestine environment, maximum values for the swelling index can be observed at 1.0%, 0.5%, and 0.25% extract dose for the SA + E, SA + P/E, and SA + G/E systems, respectively, followed by a decrease. Again, the exception is the SA + S/E matrix, where the index decreases regardless of the extract concentration used. The demonstrated relationships are based on the mechanisms described for the gastric fluid environment; however, due to the loosening of SA, a rapid release of polyphenols occurs, which also leads to higher swelling values than in the stomach [25,44]. A characteristic phenomenon that can be observed in colonic fluid is the complete disintegration of microcapsules within 8 h of incubation in the entire range of polyphenol concentrations for the SA + P/E and SA + G/E systems and for the SA + E and SA + S/E matrices from 1.5% and 1.0% of the extract dose, respectively, which is also associated with the complete release of the microcapsule core.

Based on the obtained results, it can be concluded that the selected matrices can be used as carriers of bioactive substances, demonstrating the release of polyphenolic extract from rose petals in a pH-dependent manner during digestion in the gastrointestinal tract.

### 3.6. Biocompatibility of Microcapsules

The basic requirement for the approval of new preparations and products for industrial marketing is confirmation of their safety for consumers. One method for assessing the in vitro cytotoxicity of microcapsules is their incubation in contact with a monolayer of normal cells, which serves as a model of the biological barrier. In this study, we selected a human colonocyte cell line (CCD841 CoN) for this purpose.

**Figure 5:**
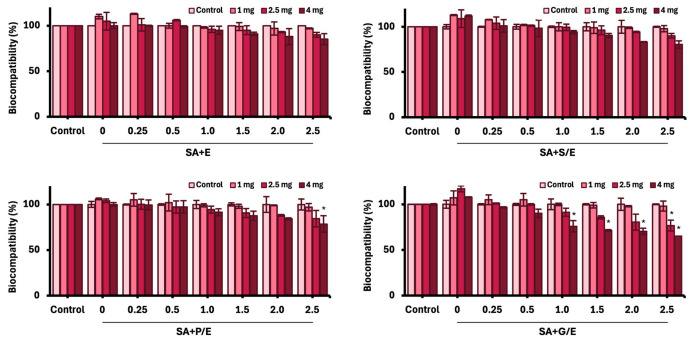
Biocompatibility of SA, SA + S, SA + P, and SA + G microcapsules with added rose petal polyphenolic extract (0.25–2.5%) against human colonocytes (CCD841 CoN cell line). Microcapsules were tested at three concentrations (1.0, 2.5, 4.0 mg/mL). Results are presented as mean and SD. Values marked with (*) are statistically significantly different compared to the control (*p* < 0.05).

As can be seen in Figure 5, for all SA, SA + S, SA + P, and SA + G matrices without the addition of rose petal extract, maintaining contact with cells at a concentration of 1.0–4.0 mg/mL, cell viability was maintained at a level of 104.0–117.3%, which indicates the lack of toxicity and cell growth promotion of the polymers used in this work. The high biocompatibility of these materials with human tissues has also been previously demonstrated [3,4,22,41]. Among others, for SA + G microcapsules kept in contact with RAW 264.7 cells (murine macrophage line), cell viability above 90% was demonstrated, which was also confirmed in in vivo studies [45].

However, the toxicity of microcapsules with a rose petal extract core towards cells depended primarily on the extract dose used and, consequently, the total polyphenolic compound content in the microcapsules. Hence, the lowest cytotoxicity was demonstrated for the SA + E system, and the highest for the SA + G/E system, consistent with the results presented in Section 3.3. Analyzing all types of extract-enriched matrices, incubation of microcapsules at a dose of 1 mg did not affect the viability of the selected cell line. In addition, no significant differences compared to the control were observed for concentrations of 2.5 and 4 mg/mL for SA + E microcapsules or for SA + S/E microcapsules tested at a concentration of 2 mg/mL with a core dose ranging from 0.25 to 2.5%. However, it is generally accepted that a dose of the preparation reducing cell viability by up to 20% can be classified as safe [46]. Hence, only SA + P/E2.5 microcapsules incubated at a dose of 4 mg/mL (inhibiting viability by 21.5%), SA + G/E2.5 microcapsules at a dose of 2.5 mg/mL (23.3%), and SA + G/E1.0–SA + G/E2.5 microcapsules at a dose of 4 mg/mL (24.2–34.8%) did not meet the above condition. This effect is likely related to the toxic effects of polyphenolic compounds in rose petals. Analyzing our results, the total polyphenol content in the above-selected microcapsules, taking into account the incubation dose, ranged from 200.3 to 320.4 μg GAE/mL. The toxic dose of polyphenolic extract from rose petals, leading to 50% inhibition of CCD841 CoN cell viability (IC50), was 484.0 μg/mL, while the total polyphenol content in this dose was 187.3 μg/mL. This relationship can therefore be explained by the toxic effect of selected microcapsules on human colonocyte cells. The same conclusions were previously reached by Żurek et al. [4] for a polyphenol fraction isolated from cranberry fruit microencapsulated in an SA + B matrix. In the aforementioned study, the toxic effect of the microcapsules was also strongly dependent on the dose of the polyphenol preparation and its effect on cell viability.

Therefore, according to this analysis, the selected microcapsules can be considered safe for oral use, with the exception of the SA + P/E2.5 and SA + G/E2.5 systems administered at high doses. However, studies using in vivo models are necessary to confirm this.

### 3.7. PCA

**Figure 6:**
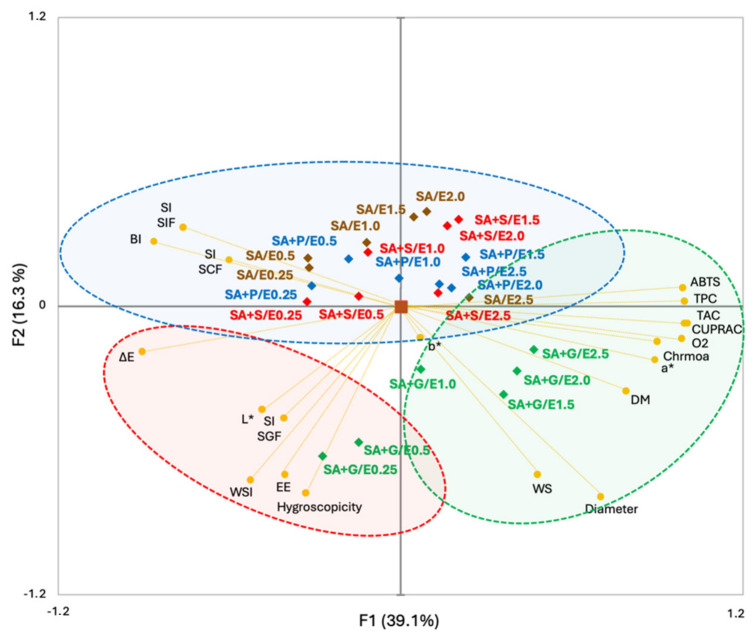
PCA between the obtained microcapsules and the determined parameters.

The relationships between the analyzed parameters and the evaluation of the diversity of the obtained microcapsules were performed using principal component analysis (PCA). The two principal components (PC1 and PC2) explained 39.1% and 16.3% of the total data variability, respectively, which accounted for 55.4% (Figure 6). The obtained variance does not fully explain all the observed differences between the tested microcapsule variants. However, the distribution of points on the graph indicates a differentiation of the samples depending on the degree of addition of polyphenolic extract from rose petals and the type of matrix used for microcapsule production. Samples composed of the SA + G matrix with the extract addition ranging from 1.0 to 2.5% were located in the lower right part of the graph, demonstrating a relationship with the total polyphenol content (TPC) and total anthocyanin content (TAC), vectors responsible for antioxidant activity (ABTS, CUPRAC, O_2_^•−^), color saturation (Chroma), the a* parameter, dry matter (DM), microcapsule diameter, and WS. The remaining microcapsules, also composed of the SA + G system (0.25%, 0.5%), constituted a separate group located in the lower left part of the graph and correlated with parameters such as L*, the gastric swelling index (SI SGF), encapsulation efficiency (EE), Δ*E*, WSI, and hygroscopicity. However, the vectors of the remaining microcapsule versions were located in the opposite direction, occupying an intermediate position in the upper part of the graph, which may indicate the lesser nature of their properties. The parameters closest to this group of samples were the swelling index in the small intestine environment (SI SIF), the colon environment (SI SCF), and biocompatibility. The obtained results are consistent with the data presented in the previous chapters. However, based on the PCA, it can be concluded that the matrix with the most favorable characteristics of the analyzed parameters was the SA + G system, and for this matrix, the results also varied most significantly depending on the extract dose used.

## 4. Conclusions

The novelty of this study was the comprehensive comparison of the effect of varying the core dose of polyphenolic extract from rose petals and various carrier systems based on modifications of sodium alginate with starch, protein, and vegetable gum on the physicochemical and functional properties of microcapsules. According to the obtained results, the SA + G matrix was the most favorable system for binding polyphenolic compounds and anthocyanins, which translated into high antioxidant activity. The dose of polyphenolic extract from rose petals that achieved the highest process efficiency was 0.25% for all matrices. Further increases in dose led to a decrease in this parameter. The use of different extract doses also affected the physicochemical properties of the microcapsules, including increases in dry weight, diameter, color parameters, and water solubility with increasing extract dose. Furthermore, across the various matrices, the highest values of these physicochemical parameters were observed primarily for microcapsules formed from the SA + G matrix. The swelling index in both gastric, intestinal, and colonic fluids decreased with increasing dose, with the highest observed for the SA + G system in gastric fluid and SA in both intestinal and colonic fluids. This differentiation in microcapsule properties was also reflected in the assessment of biocompatibility with human colonocyte membranes, where a safe dose for the SA/E and SA + S/E systems was 4 mg, and for the SA + P/E and SA + G/E systems, 2.5 and 1 mg, respectively.

The presented results provide valuable and missing data for the food and pharmaceutical industries regarding scaling the ionotropic microencapsulation process based on carrier composition and core dose. However, before industrial use, in vivo studies should be included to fully verify the health-promoting efficacy of the resulting microcapsules, as well as stability and storage stability studies that consider the impact of environmental conditions on the quality of the final product.

## Figures and Tables

**Table 1:** Physicochemical parameters of control microcapsules and microcapsules with polyphenol extract from rose petals.

Type of Microcapsules	Dry Mater	Water Solubility	Water Absorption Index	Hygroscopicity	Microcapsule Diameter	L*	a*	b*	Chroma	Δ*E*
	%	%		%	μm					
SA	90.0 ± 0.0 a	26.1 ± 0.5 e	3.2 ± 0.1 f	47.4 ± 0.2 h	388.8 ± 12.8 b	70.6 ± 0.2 k	0.6 ± 0.0 b	6.4 ± 0.2 h	-	-
SA/E0.25	91.2 ± 0.1 b	27.3 ± 0.7 ef	3.1 ± 0.1 f	46.7 ± 0.4 h	410.4 ± 10.2 c	50.3 ± 0.3 g	5.5 ± 0.7 f	0.3 ± 0.2 b	5.5 ± 0.3 a	12.6 ± 0.4 h
SA/E0.5	91.6 ± 0.1 b	29.8 ± 0.2 f	3.0 ± 0.2 e	45.9 ± 0.2 g	481.2 ± 21.2 f	50.9 ± 1.0 g	7.4 ± 1.6 g	−3.6 ± 0.8 a	8.2 ± 0.2 b	13.0 ± 0.2 h
SA/E1.0	92.4 ± 0.0 bc	30.1 ± 0.2 f	2.9 ± 0.0 d	45.0 ± 0.3 g	551.9 ± 16.2 g	49.0 ± 0.3 f	8.5 ± 0.5 gh	−2.8 ± 0.3 a	8.9 ± 0.5 bc	10.8 ± 0.1 g
SA/E1.5	92.9 ± 0.1 c	30.9 ± 0.4 f	2.9 ± 0.1 d	43.1 ± 0.3 f	563.8 ± 10.3 h	48.3 ± 0.1 e	9.8 ± 0.2 h	5.3 ± 0.1 g	11.1 ± 0.1 d	8.0 ± 0.3 f
SA/E2.0	93.2 ± 0.0 cd	31.8 ± 0.9 g	2.8 ± 0.3 d	41.4 ± 0.1 d	551.3 ± 22.4 g	44.5 ± 0.4 c	13.2 ± 0.3 k	2.1 ± 0.3 d	13.4 ± 0.2 f	2.8 ± 0.2 b
SA/E2.5	93.8 ± 0.0 d	34.2 ± 0.8 i	2.4 ± 0.0 b	37.6 ± 0.2 c	721.2 ± 43.3 j	43.1 ± 0.5 bc	13.9 ± 0.4 l	2.5 ± 0.2 d	14.1 ± 0.4 g	1.6 ± 0.3 a
SA + S	91.3 ± 0.1 b	17.3 ± 0.1 a	3.6 ± 0.2 g	52.2 ± 0.5 j	650.5 ± 20.5 i	75.7 ± 0.2 l	−0.3 ± 0.0 b	12.1 ± 0.1 i	-	-
SA + S/E0.25	92.3 ± 0.1 bc	18.5 ± 0.1 ab	3.2 ± 0.0 f	50.1 ± 0.4 i	769.0 ± 16.9 k	46.3 ± 0.5 d	3.7 ± 0.1 d	4.8 ± 0.0 fg	6.1 ± 0.1 a	12.2 ± 0.1 h
SA + S/E0.5	92.7 ± 0.0 c	20.7 ± 0.5 c	2.8 ± 0.1 d	46.8 ± 0.4 h	772.3 ± 31.3 k	42.8 ± 0.1 b	4.7 ± 0.0 ef	4.2 ± 0.0 f	6.3 ± 0.0 a	10.8 ± 0.1 g
SA + S/E1.0	92.8 ± 0.0 c	21.3 ± 0.3 d	2.7 ± 0.2 c	44.0 ± 0.3 f	901.4 ± 44.0 n	39.9 ± 0.1 a	5.6 ± 0.0 f	2.8 ± 0.0 d	6.3 ± 0.1 a	10.3 ± 0.1 g
SA + S/E1.5	93.0 ± 0.1 cd	22.8 ± 0.6 d	2.6 ± 0.0 b	37.6 ± 0.2 c	883.3 ± 41.4 m	39.0 ± 0.1 a	9.7 ± 0.0 h	2.2 ± 0.0 d	9.9 ± 0.1 c	7.1 ± 0.1 e
SA + S/E2.0	93.9 ± 0.1 d	25.4 ± 0.4 e	2.6 ± 0.1 b	36.6 ± 0.3 c	885.4 ± 37.0 m	38.2 ± 0.8 a	11.7 ± 0.4 i	1.3 ± 0.0 cd	11.8 ± 0.2 d	6.4 ± 0.2 d
SA + S/E2.5	94.4 ± 0.1 e	26.6 ± 0.8 e	2.3 ± 0.1 a	27.7 ± 0.2 b	789.4 ± 12.9 l	38.1 ± 0.4 a	12.2 ± 0.5 j	0.9 ± 0.0 bc	12.2 ± 0.2 de	6.3 ± 0.2 d
SA + P	91.1 ± 0.1 b	16.7 ± 0.2 a	2.8 ± 0.2 d	41.6 ± 0.2 e	455.2 ± 11.3 d	68.9 ± 0.0 j	1.7 ± 0.0 c	15.3 ± 0.1 j	-	
SA + P/E0.25	91.4 ± 0.0 b	17.7 ± 0.1 a	2.7 ± 0.1 c	41.2 ± 0.5 d	456.2 ± 25.5 d	53.3 ± 0.0 h	8.4 ± 0.3 gh	3.1 ± 0.0 e	9.0 ± 0.2 bc	12.4 ± 0.1 h
SA + P/E0.5	91.7 ± 0.1 b	18.1 ± 0.5 ab	2.7 ± 0.1 c	40.4 ± 0.3 d	353.4 ± 18.4 a	47.8 ± 0.0 e	9.5 ± 0.0 h	0.9 ± 0.0 bc	9.5 ± 0.1 c	7.9 ± 0.2 ef
SA + P/E1.0	92.5 ± 0.2 c	19.3 ± 0.8 b	2.6 ± 0.0 b	39.4 ± 0.2 d	367.9 ± 31.6 a	47.4 ± 0.0 de	9.6 ± 0.0 h	0.6 ± 0.0 b	9.6 ± 0.1 c	7.6 ± 0.2 ef
SA + P/E1.5	94.0 ± 0.0 d	20.1 ± 0.3 c	2.5 ± 0.3 b	37.5 ± 0.1 c	458.9 ± 36.4 de	47.2 ± 0.1 de	12.6 ± 0.2 j	0.3 ± 0.1 b	12.6 ± 0.1 e	5.7 ± 0.1 cd
SA + P/E2.0	94.3 ± 0.1 e	20.9 ± 0.3 c	2.3 ± 0.2 a	30.3 ± 0.2 b	464.5 ± 22.6 e	46.1 ± 0.2 d	12.9 ± 0.4 j	0.5 ± 0.0 b	12.9 ± 0.2 ef	4.7 ± 0.2 c
SA + P/E2.5	94.8 ± 0.0 ef	21.1 ± 0.6 d	2.2 ± 0.0 a	23.0 ± 0.2 b	469.2 ± 26.4 e	44.5 ± 0.3 c	13.3 ± 0.3 k	0.9 ± 0.3 bc	13.3 ± 0.3 f	3.3 ± 0.3 b
SA + G	92.8 ± 0.2 c	29.3 ± 0.4 f	4.7 ± 0.3 j	72.1 ± 0.4 n	887.5 ± 76.9 m	69.8 ± 0.2 j	−1.4 ± 0.3 a	5.5 ± 0.3 g	-	-
SA + G/E0.25	93.4 ± 0.1 cd	30.7 ± 0.5 f	4.4 ± 0.2 i	65.5 ± 0.4 m	903.2 ± 71.0 n	56.7 ± 0.4 i	8.1 ± 0.1 gh	2.9 ± 0.1 d	8.6 ± 0.2 b	15.4 ± 0.2 j
SA + G/E0.5	93.9 ± 0.2 d	33.1 ± 0.3 h	4.2 ± 0.2 h	63.0 ± 0.3 l	913.9 ± 64.2 o	56.0 ± 0.3 i	9.5 ± 0.1 h	1.0 ± 0.0 c	9.6 ± 0.2 c	14.3 ± 0.1 i
SA + G/E1.0	94.2 ± 0.1 e	35.2 ± 0.8 i	3.3 ± 0.1 f	61.2 ± 0.1 k	923.4 ± 55.8 p	49.8 ± 0.1 fg	12.5 ± 0.1 j	1.6 ± 0.1 cd	12.6 ± 0.2 e	7.5 ± 0.1 e
SA + G/E1.5	94.8 ± 0.1 ef	38.0 ± 0.7 j	3.0 ± 0.1 e	60.8 ± 0.2 k	954.6 ± 80.4 r	47.8 ± 0.0 e	13.8 ± 0.0 kl	2.1 ± 0.1 d	14.0 ± 0.1 g	5.1 ± 0.2 c
SA + G/E2.0	95.2 ± 0.1 f	38.9 ± 0.7 j	2.9 ± 0.0 d	51.2 ± 0.2 ij	989.9 ± 69.3 s	45.0 ± 0.3 c	13.9 ± 0.3 l	2.1 ± 0.2 d	14.1 ± 0.3 g	2.6 ± 0.1 b
SA + G/E2.5	96.0 ± 0.0 g	42.1 ± 0.9 k	2.7 ± 0.1 c	42.1 ± 0.1 ef	990.5 ± 77.3 s	40.7 ± 0.4 a	14.7 ± 0.2 m	1.5 ± 0.3 cd	14.8 ± 0.2 h	3.0 ± 0.2 b
E	99.1 ± 0.1 h	99.2 ± 0.5 l	-	5.9 ± 0.0 a	-	43.1 ± 0.1 b	15.4 ± 0.1 n	3.1 ± 0.1 e	15.7 ± 0.1 i	-

Explanations: SA, sodium alginate; S, potato starch; P, pea protein isolate; G, guar gum; E, rose hip polyphenol preparation; 0.25–2.5, extract concentrations. Results are presented as mean and SD. Values marked with different symbols (a–s) in columns differ statistically significantly (*p* < 0.05).

**Table 2:** Total polyphenol content (TPC), total anthocyanin content (TAC) and antioxidant activity (expressed by ABTS, CUPRAC, O_2_^•−^ methods) of microencapsulated polyphenolic extract from rose petals.

Type of Microcapsules	TPC	TAC	ABTS	CUPRAC	O_2_^•−^
	mg GAE/g	mg C3G/g	mmol TE/100 g		μg/mL
SA	0.0 ± 0.0 a	0.0 ± 0.0 a	0.0 ± 0.0 a	0.0 ± 0.0 a	>1000
SA/E0.25	14.4 ± 0.0 b	1.1 ± 0.0 b	8.0 ± 0.0 b	2.1 ± 0.0 b	>1000
SA/E0.5	24.0 ± 0.0 c	2.1 ± 0.0 c	27.1 ± 0.1 c	7.3 ± 0.0 c	>1000
SA/E1.0	37.4 ± 0.1 e	3.1 ± 0.0 d	48.4 ± 0.1 f	13.2 ± 0.0 e	902.2 ± 1.6 h
SA/E1.5	47.6 ± 0.2 f	4.3 ± 0.1 e	70.4 ± 0.1 h	21.7 ± 0.1 h	715.6 ± 2.1 g
SA/E2.0	58.6 ± 0.1 h	5.0 ± 0.1 f	85.0 ± 0.2 i	24.5 ± 0.1 i	569.1 ± 0.9 d
SA/E2.5	66.8 ± 0.2 j	5.8 ± 0.0 h	94.0 ± 0.1 j	25.8 ± 0.1 i	509.5 ± 1.5 b
SA + S	0.0 ± 0.0 a	0.0 ± 0.0 a	0.3 ± 0.0 a	0.1 ± 0.0 a	>1000
SA + S/E0.25	16.9 ± 0.0 b	1.3 ± 0.0 b	8.0 ± 0.0 b	2.3 ± 0.0 b	>1000
SA + S/E0.5	24.9 ± 0.0 c	2.2 ± 0.0 c	27.5 ± 0.0 c	7.6 ± 0.0 c	>1000
SA + S/E1.0	37.3 ± 0.1 e	3.2 ± 0.0 d	50.4 ± 0.1 f	14.5 ± 0.0 f	904.8 ± 1.7 h
SA + S/E1.5	53.0 ± 0.2 g	4.5 ± 0.1 e	84.8 ± 0.2 i	25.8 ± 0.1 i	641.8 ± 1.2 f
SA + S/E2.0	59.0 ± 0.0 h	5.2 ± 0.1 g	93.5 ± 0.2 j	28.2 ± 0.1 j	566.5 ± 0.9 d
SA + S/E2.5	64.3 ± 0.1 i	5.9 ± 0.1 h	103.2 ± 0.2 k	30.3 ± 0.2 jk	535.8 ± 1.0 c
SA + P	0.0 ± 0.0 a	0.0 ± 0.0 a	0.7 ± 0.0 a	0.1 ± 0.0 a	>1000
SA + P/E0.25	19.5 ± 0.0 b	1.5 ± 0.0 b	8.2 ± 0.0 b	2.5 ± 0.0 b	>1000
SA + P/E0.5	27.3 ± 0.1 d	2.4 ± 0.0 c	29.6 ± 0.1 d	8.1 ± 0.0 cd	>1000
SA + P/E1.0	37.6 ± 0.1 e	3.3 ± 0.0 d	59.7 ± 0.1 g	16.7 ± 0.1 g	899.0 ± 2.2 h
SA + P/E1.5	55.5 ± 0.1 g	4.7 ± 0.0 ef	86.9 ± 0.2 i	27.7 ± 0.1 ij	608.1 ± 2.0 e
SA + P/E2.0	64.2 ± 0.1 i	5.7 ± 0.1 h	104.8 ± 0.3	31.9 ± 0.1 k	538.8 ± 2.1 c
SA + P/E2.5	68.3 ± 0.0 j	6.0 ± 0.0 h	116.7 ± 0.3 l	36.4 ± 0.0 l	499.3 ± 0.9 b
SA + G	0.0 ± 0.0 a	0.0 ± 0.0 a	0.9 ± 0.0 a	0.2 ± 0.0 a	>1000
SA + G/E0.25	26.2 ± 0.1 cd	2.3 ± 0.0 c	8.2 ± 0.1 b	2.6 ± 0.0 b	>1000
SA + G/E0.5	35.7 ± 0.0 e	3.3 ± 0.0 d	39.9 ± 0.1 e	10.9 ± 0.0 d	946.9 ± 1.7 i
SA + G/E1.0	54.7 ± 0.0 g	4.9 ± 0.1 f	71.1 ± 0.2 h	22.2 ± 0.0 h	619.1 ± 1.3 ef
SA + G/E1.5	67.2 ± 0.1 j	5.9 ± 0.1 h	108.1 ± 0.3 k	41.2 ± 0.1 m	501.4 ± 1.3 b
SA + G/E2.0	74.1 ± 0.2 k	6.6 ± 0.1 i	119.3 ± 0.3 l	43.1 ± 0.1 mn	455.6 ± 1.0 a
SA + G/E2.5	80.1 ± 0.2 l	7.2 ± 0.1 j	133.3 ± 0.3 m	46.7 ± 0.1 n	428.4 ± 0.8 a
E	387.0 ± 0.3 m	32.1 ± 0.2 k	763.4 ± 0.8 n	285.2 ± 0.4 o	87.4 ± 0.3 a

Explanations: SA, sodium alginate; S, potato starch; P, pea protein isolate; G, guar gum; E, rose hip polyphenol preparation; 0.25–2.5, extract concentrations. Results are presented as mean and SD. Values marked with different symbols (a–o) in columns differ statistically significantly (*p* < 0.05).

## Data Availability

The original contributions presented in this study are included in the article/Supplementary Material. Further inquiries can be directed to the corresponding author.

## Supplementary Materials

The following supporting information can be downloaded at: https://www.mdpi.com/article/10.3390/foods15122134/s1, Figure S1. Effect of polyphenolic extract from rose petals on the viability of human colonocytes (CCD841 CoN cell line).
