# Supplementary material for: Physicochemical and Functional Properties of Polyphenolic Core Gel Microcapsules from Rose Petals (Rose L.): A Comparative Study
Source: Foods. 2026 Jun 13;15(12):2134. doi: 10.3390/foods15122134 (PMC13297839; doi:10.3390/foods15122134)
Supplement: Supplementary file 1 [file foods-15-02134-s001.zip › foods-4352658-supplementary.pdf]

Supplementary material

Physicochemical and functional properties of polyphenolic core gel microcapsules from rose petals (*Rose* L.): a comparative study

Natalia Żurek [1,*], Andżelika Padowska[1], Andżelika Kusy[1] and Karolina Ochab[1]

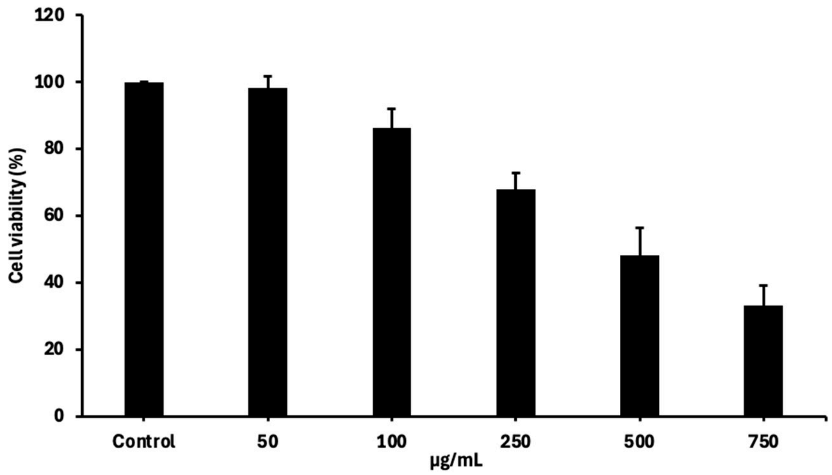

Figure S1. Effect of polyphenolic extract from rose petals on the viability of human colonocytes (CCD841 CoN cell line).
